# Advances in genetic abnormalities, epigenetic reprogramming, and immune landscape of intracranial germ cell tumors

**DOI:** 10.1186/s40478-023-01682-y

**Published:** 2023-11-27

**Authors:** Yi Zhang, Chengyi Zhong, Xindi Ke, Jifang Liu, Zhang Ye, Lin Lu, Kan Deng, Huijuan Zhu, Yong Yao

**Affiliations:** 1 Department of Neurosurgery, Peking Union Medical College Hospital, Chinese Academy of Medical Sciences and Peking Union Medical College, Beijing, 100730 China; 2 Chinese Academy of Medical Sciences and Peking Union Medical College, Beijing, 100730 China; 3 Department of Endocrinology, Peking Union Medical College Hospital, Chinese Academy of Medical Sciences and Peking Union Medical College, Beijing, 100730 China

**Keywords:** Intracranial germ cell tumors (IGCTs), Biomarkers, Genetic abnormality, Epigenetic reprogramming, Immune landscape

## Abstract

Intracranial germ cell tumors (IGCTs) are a rare subtype of central nervous system neoplasms that predominantly affect young individuals and exhibit a higher incidence in East Asia. IGCTs can be pathologically divided into two main categories: germinomas and non-germinomatous germ cell tumors (NGGCTs). Despite the scarcity of this disease, recent advancements in molecular biology techniques have facilitated the discovery of the inherent genetic and molecular characteristics of IGCTs. Somatic mutations that result in the activation of the KIT/RAS/MAPK and PI3K/AKT/mTOR pathways, chromosomal instability leading to characteristic changes in chromosomal fragments (notably 12p gain), and potentially diagnostic miRNAs (such as miR-371a-3p) may provide valuable insights for the efficient diagnosis, targeted therapy, and prognosis evaluation of IGCTs. Additionally, transcriptomic and methylomic analyses have provided new perspectives on the intrinsic development of IGCTs, further elucidating their equivalence with GCTs at other sites. The evaluation of the tumor immune landscape may guide prognosis prediction and immunotherapy for IGCT patients. Nevertheless, current research still faces challenges such as the absence of basic laboratory research systems, a single source of large sample research data, and a limited overall volume of research. The incorporation of larger sample sizes, the implementation of more innovative evaluation systems, and the employment of novel experimental methods are urgently required to become the focus of future research.

## Introduction

Intracranial germ cell tumors (IGCTs) or central nervous system germ cell tumors (CNS GCTs) are a rare subtype of extragonadal germ cell tumors (GCTs) that arise within the central nervous system (CNS). These tumors are histologically indistinguishable from GCTs located elsewhere within the body, yet there is no evidence of a primary tumor within the gonads. IGCTs predominantly affect children and young adults, with a median age at diagnosis between 10 and 14 years [6] and a male-to-female ratio ranging from 2:1 to 3:1 [10]. The incidence of IGCTs varies greatly worldwide, with rates in East Asia being 3–8 times higher than those observed in Western countries. In Asia, GCTs account for 10–15% of all primary pediatric CNS tumors [5], while in the United States and Europe this figure falls to only 1–3% [21].

The 5th edition of the WHO Classification of Tumors of the Central Nervous System, published in 2021, categorizes IGCTs histologically into germinomas, mature teratomas, immature teratomas, teratomas with somatic-type malignancy, embryonal carcinomas, yolk sac tumors, choriocarcinomas, and mixed GCTs [16]. Germinomas account for 60–65% of all pediatric IGCTs [10] and IGCTs other than germinomas are collectively referred to as non-germinomatous germ cell tumors (NGGCTs). NGGCTs secrete alpha-fetoprotein (AFP), mainly produced by yolk sac tumors and beta-human chorionic gonadotropin (β-HCG), which can be secreted by choriocarcinoma. These markers can be detected in serum or cerebrospinal fluid (CSF). Germinomas do not secrete AFP but certain components within them can secrete small amounts of HCG. Serum/CSF AFP/HCG levels serve as important tumor markers for distinguishing the histological types of IGCTs in clinical practice [2]. Germinomas exhibit sensitivity to both radiotherapy and platinum-based chemotherapy. In contrast to germinomas, NGGCTs show poorer outcomes with radiotherapy alone, but are sensitive to chemotherapy, thus making combination therapy a promising approach. Compared to germinomas, NGGCTs have a relatively poorer prognosis [18].

IGCTs primarily occur in midline structures, with the most common sites of origin being the pineal (50%) and suprasellar region (20–30%). Other potential sites of origin include the third ventricle, basal ganglia, and thalamus [15, 23]. The clinical presentation of IGCTs is closely related to both tumor location and size. For instance, IGCTs located within the pineal region often result in increased intracranial pressure due to obstruction of CSF circulation. If the tumor compresses the tectum, it may also induce Parinaud’s syndrome [5, 24]. IGCTs located within the suprasellar region most commonly result in hypothalamic-pituitary dysfunction, manifesting clinically as various types of endocrine disorders such as diabetes insipidus, growth retardation, precocious puberty, adrenal insufficiency, and hypothyroidism [5, 23]. IGCTs located within the basal ganglia typically result in pyramidal system dysfunction and may present as progressive mild hemiparesis [12].

Despite the low incidence of IGCTs and significant variation in incidence rates worldwide, there exists a relatively small body of basic research on IGCTs, with a considerable portion originating from Japan. In recent years, with the rapid development of molecular biology techniques such as next-generation sequencing (NGS), research into the pathogenesis and pathological characteristics of IGCTs has been actively pursued. Genomic mutation spectrum and copy number analyses have revealed the importance of the KIT/RAS/MAPK and PI3K/AKT/mTOR pathways in the pathogenesis of IGCTs [25]. Transcriptomic and epigenetic studies have elucidated the differentiation characteristics and potential developmental origins of different IGCT subtypes. Concurrently, analyses of non-coding RNAs have proposed potentially more reliable detection indicators while providing possible explanations for the distinct biological characteristics exhibited by different IGCT subtypes. Additionally, research into the tumor immune microenvironment (TIME) of IGCTs provides predictive insights for targeted tumor therapy and prognosis from another perspective. This article summarizes current basic research achievements in IGCTs from the perspectives of somatic mutations, chromosomal variations, transcriptomic characteristics (including non-coding RNAs), epigenetic reprogramming, and immune microenvironment while highlighting deficiencies within this field of research and proposing potential future research directions.

## Somatic mutation

The initial speculation regarding the presence of *KIT* mutations in IGCTs arose from the observation that almost all testicular seminomas/non-seminomas exhibit KIT membrane staining, with a considerable portion harboring *c-kit* mutations [11, 26]. Utilizing polymerase chain reaction (PCR) and Sanger's sequencing, a research team from Japan first confirmed the presence of *c-kit* mutations in IGCTs, although these mutations were not related to clinicopathological differences [27]. To further explore the biological rationale and impact on patient survival of *KIT* mutations, Fukushima et al. conducted a study on multiple genes within the KIT signaling pathway in 65 IGCT samples. Results indicated that mutations were present only in *KIT* and its downstream gene *RAS*, with all *KIT* and *RAS* mutations being mutually exclusive. Compared to NGGCTs, germinomas exhibited a higher KIT positivity rate and more frequent *KIT* mutations. Correlation studies revealed that for germinomas, overexpression of KIT was largely related to *KIT/RAS* mutations, with the *KIT/RAS* mutation status exerting a significant adverse effect on patient prognosis [3]. Subsequently, Wang et al. applied NGS to the analysis of IGCT cases for the first time, identifying many other genetic changes in 62 cases besides *KIT*. Data indicated that more than half of the tumors carried at least one somatic mutation in genes involved in either the KIT/RAS or AKT/mTOR pathway [37]. A subsequent study compared 124 IGCTs with 65 testicular germ cell tumors (TGCTs) and 8 GCTs that had metastasized to the CNS. In addition to confirming previous conclusions, this study demonstrated that mutated *mTOR* resulted in upregulation of PI3K pathway signaling and enhanced cell migration. Germinomas and NGGCTs, as well as IGCTs and TGCTs, all exhibited similar mutation characteristics, suggesting that regardless of site of origin, the main molecular pathogenesis of various GCTs involves somatic point mutation activation of either the MAPK and/or PI3K/mTOR pathway [9]. However, a comprehensive analysis of 190 IGCT cases revealed that IGCTs with MAPK pathway mutations and PI3K/mTOR pathway mutations exhibit different intracranial sites of origin, with MAPK pathway mutations being significantly more common in male cases [32]. Recently, a genome-wide associated study revealed that susceptibility loci for germline mutations are also present in cases of IGCTs. Specifically, a 4-bp deletion in the enhancer adjacent to the BAK1 gene is associated with an increased risk of IGCTs. This association is independent of ethnicity and the primary site of GCTs [30].

**Fig. 1:**
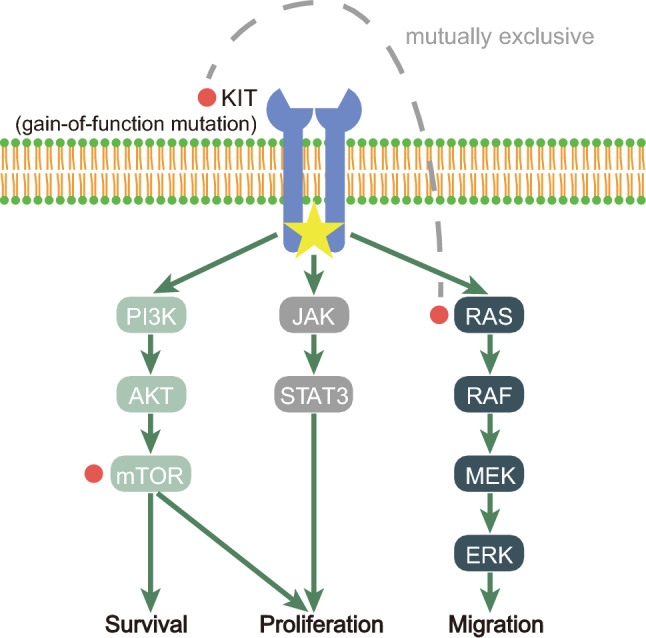
Pathways involved in common mutations in IGCTs. The red solid circles represent the most typical mutated genes in the pathway

In summary, current research indicates that the genetic driving factors of IGCTs pathogenesis primarily involve the KIT/RAS/MAPK and PI3K/AKT/mTOR pathways. Gene mutations within the MAPK pathway, such as *KIT* and *RAS*, are mutually exclusive, while *mTOR* represents the most common mutation within the PI3K/AKT/mTOR pathway (Fig. 1). Mutations in both pathways ultimately result in activation of their respective pathways. GCTs of different origins exhibit similar molecular pathogenesis. However, variations in the MAPK or PI3K/mTOR pathways may contribute to the differences in intracranial origin sites of IGCTs. For instance, mutations in the PI3K/mTOR pathway are more common in basal ganglia lesions [32].

## Chromosomal aberrations

Analysis of chromosomal variations is often closely related to research on somatic mutations. In their study of KIT signaling pathway mutations, Fukushima et al. also classified the chromosomal status of IGCTs into two categories: severe instability and mild/negative instability. There was no difference in the distribution of germinomas and NGGCTs within this classification. In germinoma cases, overexpression of KIT was associated with severe chromosomal instability, with patients exhibiting an unstable chromosomal state demonstrating a significantly worse prognosis [3]. A subsequent comprehensive analysis of 190 IGCT cases revealed that basal ganglia lesions, elderly cases, and cases with PI3K/mTOR pathway mutations were concentrated within the chromosomally unstable group [32].

On the other hand, Wang et al. discovered that 18% of samples exhibited copy number gains of 14q32.33, which contains 34 genes, including *AKT1*. Gains of this segment were associated with overexpression of *AKT1* [37]. Two small-sample studies from Poland reported that chromosomal variations are common in IGCTs, with the most common numerical variations being trisomies 19 and 21 and monosomies 13 and 18. The most common structural variation was 12p gain [13, 14]. The 12p region includes key genes such as *NANOG*, *KRAS*, and *CCND2*, which play significant roles in tumor initiation and progression. Satomi et al. conducted a specific study on the phenomenon of 12p gain in 82 IGCTs, revealing that 12p gain is the most common chromosomal aberration in IGCTs and is more common in NGGCTs, particularly in their malignant components. 12p gain was associated with poor prognosis in IGCTs and was mutually exclusive with *KIT* mutations [28]. A recent study by Takami et al. found that, independent of 12p gain, the 3p25.3 gain, which is exclusive to non-germinomatous germ cell tumors (NGGCTs), can also serve as a marker of poor prognosis [35]. In summary, some IGCTs exhibit characteristics of chromosomal instability, with the most common chromosomal aberration being 12p gain, which is more prevalent in the malignant components of NGGCTs. These characteristics may indicate a poor prognosis for these patients.

## Transcriptomic characteristics

Wang et al. conducted a comparative analysis of the transcriptomes of 161 pediatric IGCTs and embryonic stem cells (ESCs). The results of supervised clustering analysis revealed significant differences between the transcriptional profiles of germinomas and non-malignant NGGCTs (NGMGCTs). Specifically, genes responsible for self-renewal and immune response were found to be highly expressed in germinomas, while genes associated with neuronal differentiation, the Wnt/β-catenin pathway, invasiveness, and epithelial-mesenchymal transition were enriched in NGMGCTs [36]. In a separate study, Takami et al. performed unsupervised clustering of the transcriptomes of 58 IGCTs and 3 TGCTs. Their results showed that the two groups could be well distinguished: the expression profile of germinomas was found to be similar to that of primordial germ cells (PGCs), while the expression profile of NGGCTs was found to be similar to that of ESCs [31]. These comparative transcriptomic studies of IGCTs provide insights into the origin from embryogenesis, differences in pathophysiology of different IGCT subtypes as well as the corresponding associations with differentiatial status.

Another focus of transcriptomic research is the role of non-coding RNAs. In a study of 66 GCTs (including IGCTs), Murray et al. discovered significant differences in microRNA (miRNA) expression between yolk sac tumors (YSTs) and germinomas. These miRNAs target transcription factors such as GATA6 and may contribute to the increased invasiveness observed in YSTs [20]. Wang et al. reported that germinomas and NGGCTs exhibited distinct miRNA expression profiles in their study of IGCT transcriptomes, with the majority of differentially expressed miRNAs being upregulated in NGMGCTs [36]. Hsieh et al. found that upregulation of miRNAs in NGMGCTs was associated with low methylation levels in their study of IGCT methylation profiles [8].

In addition to elucidating molecular biology mechanisms, miRNAs show promise as novel biomarkers. Murray et al. found that levels of miRNA-371a-3p in serum and CSF could distinguish between IGCTs and CNS Langerhans cell histiocytosis [19]. Schönberger et al. proposed additional diagnostically significant miRNAs, including the miR-371 ~ 373 and miR-302/367 clusters, which could detect expression differences in patients who tested negative for traditional biomarkers such as AFP and β-HCG [29]. Given the current limitations of biomarker-based diagnostics, specific miRNA levels in serum and CSF may serve as more efficient and precise diagnostic indicators. However, a study conducted in Singapore reported differences in CSF miRNA levels between Chinese and Malay individuals, suggesting that the use of miRNAs as an auxiliary diagnostic tool for IGCTs should take into account racial differences. The diagnostic value of miRNAs as novel markers for IGCTs will require validation by future large-scale cohort studies [17].

## Epigenetic reprogramming

Fukushima et al. conducted the first whole-genome methylation study of IGCTs. Using unsupervised clustering, the methylation characteristics of IGCTs were found to divide the tumors into three subtypes, with the methylation subtype being highly consistent with the pathological type. Notably, low methylation levels were identified as a distinct feature of germinomas, similar to PGCs. Despite the presence of identical somatic mutations in different histological components of mixed IGCTs, germinoma components and non-germinoma components exhibited significantly different methylation characteristics, with the former exhibiting low methylation levels and the latter exhibiting high methylation [4]. These results suggest that different types of IGCTs share a common origin and that epigenetic reprogramming determines their differentiation pathways. Takami et al. divided IGCTs and TGCTs into two groups based on their methylation profiles, with the primary difference between the groups being pathological type rather than lesion location. This finding suggests that the pathophysiological differences between GCTs are largely independent of their origin location and are instead determined by their differentiation state [31]. In another study, Hsieh et al. used methylation and hydroxymethylation sequencing to identify miRNAs that were differentially expressed in germinomas and NGMGCTs and were associated with gene methylation: miR-214-3p and miR-199a-5p. Cell-based experiments confirmed that demethylation could upregulate the expression of both miRNAs, and that transfection of miR-214-3p increased tumor cell resistance to cisplatin. These findings suggest that methylation levels can influence tumor cell traits by regulating miRNA expression, resulting in prognostic differences between different subtypes of IGCTs [8]. Epigenetic research not only highlights the consistency between IGCTs and GCTs from other anatomical sites but also suggests that methylation regulation plays a crucial role in determining the characteristics of IGCTs.

## Immune landscape

The TIME has emerged as a research hotspot in the study of the development, progression, and treatment of various solid tumors. In recent years, there have been advances in the study of TIME in IGCTs. Takami et al. analyzed the cellular composition of 100 germinomas and found that the expression of tumor immune-related genes varied with different tumor cell contents. High CD4 expression was significantly associated with a favorable prognosis, while an increase in nitric oxide synthase 2 was associated with an unfavorable prognosis, and most germinomas expressed PD1 [33]. In another study, researchers divided intracranial germinomas patients into two groups based on the time from symptom onset to case diagnosis: long-term onset and short-term onset. Immunohistochemical analysis showed that the former had a lower positive cell rate of PD-L1, higher levels of CD3+/CD8+ lymphocytes, and a lower ratio of PD-1+ cells/CD8+ cells, indicating that the immune microenvironment affects the growth of germinomas. This may explain to some extent the cause of phenotypic differences between germinoma groups [22]. A study from China focused on the impact of high endothelial venules (HEVs) on the immune landscape in germinomas. Through immunohistochemical staining of 42 germinomas, HEVs were detected in 31% of samples. The presence of HEVs was associated with abundant infiltration of CD3+T, CD20+B, and CD8+T cells. Higher HEV levels were associated with a favorable prognosis for germinomas [1]. Takami et al. found that NGGCTs had significantly higher levels of immune cell infiltration compared to germinomas in their transcriptomic study [31]. Research on the tumor microenvironment has deepened our understanding of tumor biology behavior in other tumor fields and provided new ideas for tumor treatment. However, compared to other tumors, there is still a gap in research on IGCTs, so it is necessary to conduct more in-depth research. For example, the relationship between the IGCT immune microenvironment and the development and prognosis of different subtypes of tumors is worthy of further study and discussion.

## Discussion

This article summarizes the basic research achievements on IGCTs over the past decade. These studies primarily focus on molecular aspects, using bioinformatics methods to correlate the intrinsic characteristics of tumors, such as genome, transcriptome, methylome, and immune characteristics, with external characteristics such as clinical pathology, providing profound insights into the occurrence and development of IGCTs.

A common consensus among various research results is that different genetic characteristics can distinguish different pathological types of IGCTs but cannot distinguish between GCTs originating from various sites. However, it is worth noting that the intracranial site of IGCT occurrence may be related to genetic characteristics. In any case, this further confirms that IGCTs are not only histologically but also essentially the same as GCTs from other sites in terms of occurrence.

The various molecular and histological characteristics found in basic research can provide a basis for more precise histological typing. At the same time, this also provides a basis for more efficient and accurate diagnosis. Research on the correlation between IGCT genetics, immunology characteristics and its development, prognosis and other aspects can provide effective guidance for predicting the clinical outcome of patients. For patients with different predicted outcomes, different treatment modalities need to be adopted in a timely manner. The study of tumor molecular mechanisms will help the development of new targeted treatment sites. For example, targeting most IGCTs with upregulated KIT/RAS/MAPK pathway and/or PI3K/AKT/mTOR pathway, designing inhibitors for these pathways may be a new idea for developing tumor-targeted drugs. For pathological subtypes with poor prognosis, differential molecular characteristics (such as transcriptional profiles, methylation profiles, etc.) can be screened or immune microenvironment characteristics identified to provide ideas for personalized treatment plans.

However, there are still many shortcomings in the research up to now. First of all, almost all research relies on sequencing of clinical specimens and lacks basic research models. Further research may require the establishment of a more mature laboratory research system, such as establishing corresponding cell lines, exploring tumor modeling schemes in vitro experiments, conducting pathway research at the cellular level and designing drug sensitivity tests.

Secondly, most of the large-scale studies currently come from Japanese teams, and the main research conclusions may have systematic errors due to regional factors. The Intracranial Germ Cell Tumor Genome Analysis Consortium of Japan (IGCT Consortium) IGCT database includes IGCT cases from 20 participating institutions since its creation [34], providing sufficient resources for large-scale data analysis and serving as a model for other countries and regions to follow. One direction for future research is to expand the cohort to include cases from more regions globally and conduct comprehensive analysis to explore the characteristic or universal laws of cases in different populations.

In addition, the combined application of single-cell sequencing and spatial transcriptomics technology can improve the resolution to the single-cell level while obtaining tissue spatial location information. This will help reveal the origin of mixed IGCTs, describe the tumor immune landscape, and other issues. More hallmarks of tumors, such as vascularization, senescent cells, polymorphic microbiomes, phenotypic plasticity, and other areas that have not yet been studied [7], can become the focus of future research. Further research directions may include tumor exosomes and cell-free DNA, which may provide more non-invasive indicators for clinical use and establish more precise and effective markers for diagnosis, treatment, and prognosis.

## Data Availability

Not applicable.
